# The impact of the 5A nursing model on pain in patients with postherpetic neuralgia: a randomized controlled trial

**DOI:** 10.3389/fpain.2026.1769480

**Published:** 2026-06-17

**Authors:** Xi Yi

**Affiliations:** Department of Pain, Zunyi Medical University Affiliated Hospital, Zunyi, China

**Keywords:** 5A nursing model, conventional nursing, pain management, postherpetic neuralgia, randomized controlled trial

## Abstract

**Background:**

The complex pathogenesis of postherpetic neuralgia significantly impacts patients' quality of life. Traditional nursing models often fail to fully meet patients’ individual needs, whereas the 5A nursing model emphasizes individualized and comprehensive care, providing patients with more effective pain management. This study aims to explore the impact of the 5A nursing model on the pain intensity of patients with postherpetic neuralgia, providing a scientific and effective nursing protocol for clinical practice.

**Methods:**

This study employed a prospective methodological design and enrolled patients aged 18 years and older who were admitted to the pain management department and fulfilled the diagnostic criteria for postherpetic neuralgia from July 2025 to October 2025. Participants were required to complete a demographic questionnaire and a numerical pain rating scale. Subsequently, 120 patients were randomly assigned to two groups: the conventional nursing group (*n* = 60) and the 5A nursing model group (*n* = 60). The Numeric Rating Scale (NRS) will be used to quantitatively assess pain levels in both groups at baseline, day 1, day 14, and one month post-intervention, facilitating a comparative analysis of the additional effect of the 5A nursing model on pain relief.

**Results:**

The baseline characteristics, including age, gender, medical history (such as diabetes and hypertension), and pain location, were statistically comparable between the conventional nursing group and the 5A nursing model group, with no significant differences observed (*P* > 0.05). However, the Numerical Rating Scale (NRS) scores for pain in the 5A nursing model group were notably lower compared to those in the conventional nursing group, indicating a statistically significant difference (*P* < 0.05).

**Conclusion:**

Nursing interventions based on the 5A model have been demonstrated to enhance pain management for patients suffering from postherpetic neuralgia, thereby improving their overall disease condition. Consequently, clinical nursing staff are encouraged to adopt this model in managing patients’ pain, and to intensify intervention measures in order to foster patients’ well-being and recovery.

**Trial registration:**

This clinical study was registered at Chinese Clinical Trial Registry (ChiCTR2500104499) with registration date of 18/6/2025.

## Introduction

Herpes zoster (HZ) disease occurs globally, with its incidence rate increasing as age advances and remaining consistent across seasons. Studies have shown that among young individuals, the annual incidence rate is between 1.2 and 3.4 cases per 1,000 people, whereas in elderly patients, this rate rises to 3.9 to 11.8 cases per 1,000 people. Similarly, a systematic review of studies conducted between 2002 and 2018 estimates the cumulative incidence of HZ to be between 2.9 and 19.5 cases per 1,000 individuals (1, 2). Postherpetic neuralgia (PHN) is the persistent pain that remains after the healing of herpes zoster rash, representing a common complication of HZ (3, 4). In China, approximately 29.8% of HZ patients develop PHN, and the prevalence rate increases with age (5). A study has found that shingles not only affects patients’ daily activity ability, physical function, and quality of life, but also has a more significant negative impact on patients’ quality of life compared to shingles patients without postherpetic neuralgia. And the longer the duration of postherpetic neuralgia, the more significant the negative impact on patients’ quality of life. Persistent pain can last for months or years, causing weakness and requiring long-term pain management (3).

Currently, postherpetic neuralgia was initially treated with medication. The European Federation of Neurological Societies has presented A-level evidence for first-line and second-line drug treatments, such as tricyclic antidepressants, pregabalin, tramadol, etc. However, this conservative treatment is not effective for all patients (6, 7). The pathogenesis of PHN is complex, and there is a lack of effective treatment methods. The traditional nursing model often employs standardized nursing procedures, but in the pain management of patients with PHN, due to significant individual variations among patients in terms of pain intensity, nature, and duration, standardized nursing procedures fail to meet the personalized needs of patients. This may lead to suboptimal nursing outcomes and ineffective relief of patients’ pain. The 5A nursing model focuses on behavior change and covers five stages: assessment, advice, agreement, assist, and arrangement. It can systematically promote patient self-management and is particularly suitable for the long course of PHN, the need for long-term medication, and lifestyle adjustments; Its structured follow-up mechanism specifies the frequency and content of follow-up in the Arrange step, which can adjust the analgesic plan in a timely manner during pain fluctuations to prevent recurrence or adverse reactions; The Agree process emphasizes the joint development of nursing plans by healthcare professionals and patients to enhance their self-efficacy and compliance. Previous studies have shown that patient participation is a key factor in successful chronic pain management; In addition, each step of 5A can be completed by multidisciplinary members such as nurses, pharmacists, and rehabilitation therapists, meeting the needs of PHN for comprehensive interventions in medication, physics, and psychology (8–10).

Currently, studies have applied the 5A nursing model to populations such as patients undergoing knee arthroplasty and those after emergency percutaneous coronary intervention, achieving promising results. While the 5A nursing model has been applied to evaluate the quality of life and self-management abilities of patients with conditions such as acute coronary syndrome, hypertension, and diabetes, researchers have yet to discover any studies examining its impact on pain management specifically for patients with postherpetic neuralgia resulting from herpes zoster (11–13). Given this gap, the present study aims to explore the effectiveness of a pain management plan based on the 5A nursing model in addressing the pain experienced by patients with postherpetic neuralgia. The objective is to determine whether this model can offer a beneficial approach to managing the often debilitating pain associated with this condition.

## Methods

### Study design and participants

We conducted this prospective randomized controlled trial from July 2025 to October 2025, selecting patients admitted to the Department of Pain Medicine who met the diagnostic criteria for postherpetic neuralgia as the study subjects. Inclusion criteria: (1) Age ≥ 18 years; (2) Admitted to our hospital's Pain Management Department; (3) PHN was defined by the presence of HZ-associated pain rated as ≥3 on an 11-point Numeric Rating Scale (NRS) (14), persisting or appearing more than 90 days after onset of the HZ rash (4). Patients have provided informed consent and voluntarily agree to participate in this study. Exclusion criteria: (1) Patients with other chronic diseases or complications that may cause pain. (2) Patients should not have severe organic diseases of the heart, lungs, liver, kidneys, etc. (3) Patients participating in other clinical studies that may affect pain perception or management.

**Figure 1:**
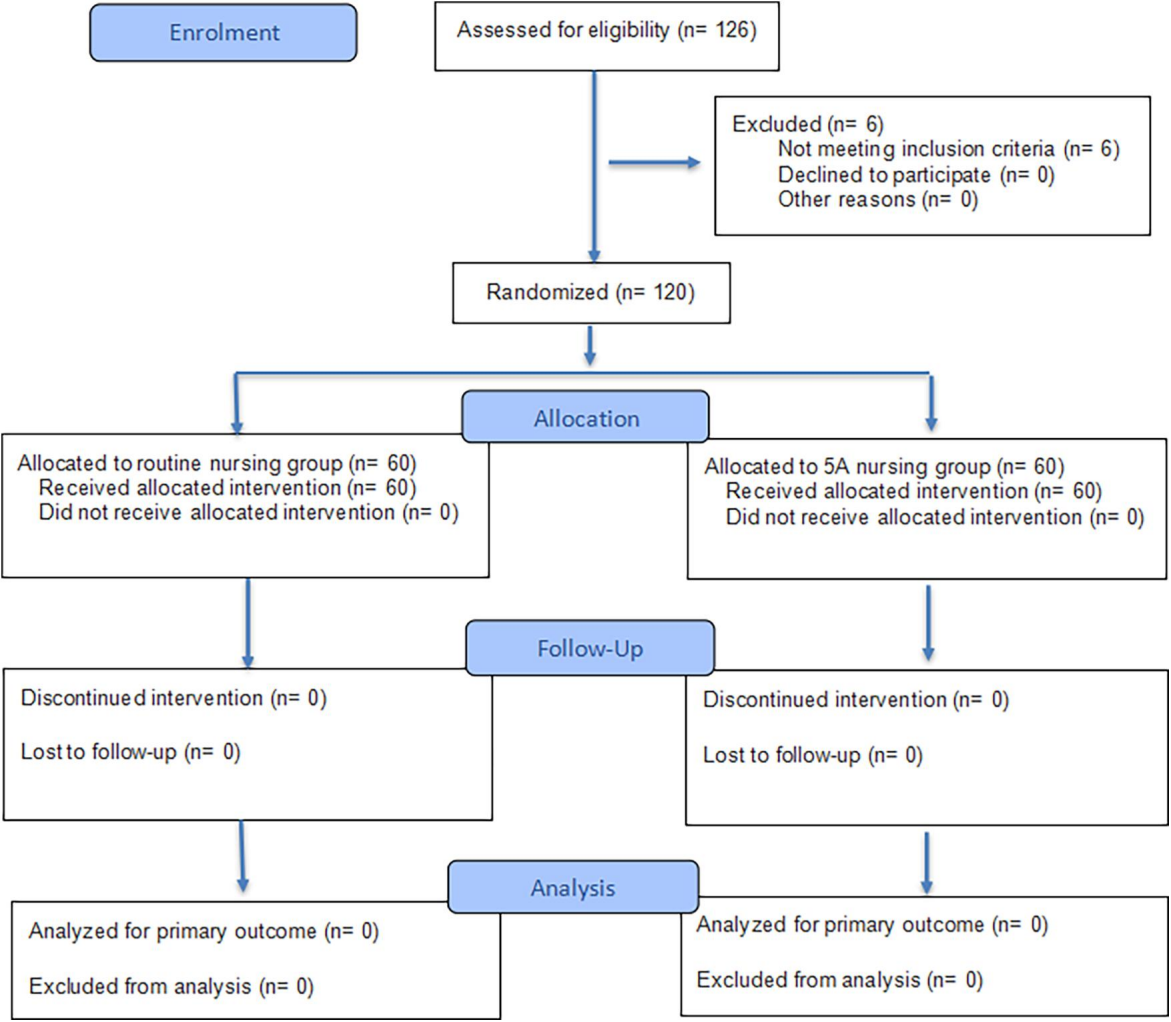
Consort flow diagram. Adapted from CONSORT 2010 Statement: updated guidelines for reporting parallel group randomised trials by Schulz KF, Altman DG, Moher D, for the CONSORT Group, licensed under CC BY, http://www.consort-statement.org/.

This study has been approved by the Ethics Committee of the Zunyi Medical University Affiliated Hospital (KLL-2024-515). All participants will sign an informed consent form, understanding the study's purpose, procedures, and potential risks and benefits. Patient privacy will be strictly protected throughout the study, and all data will be used solely for research purposes, ensuring confidentiality and security. The procedures were conducted in accordance with the ethical standards set forth by the Committee on Human Experimentation and the Helsinki Declaration of 1964, as revised in 2013. Moreover, this randomized controlled trial was conducted and reported following the CONSORT guidelines (Figure 1).

### Randomization procedure

This study used a random-number table method for group assignment. Each participant received a unique random number at enrollment. Participants with odd numbers were assigned to the control group, and participants with even numbers were assigned to the intervention group. The allocation information was recorded and retained by two independent investigators to ensure transparency and traceability.

### Blinding and bias management

Participants were aware of their allocated treatment because the nature of the intervention (the 5A nursing model) could not be masked. This inevitably introduced a potential expectation bias (participants in the intervention group might expect better outcomes due to increased attention). To mitigate implementation bias, the intervention was delivered strictly according to a standardized protocol (SOP) by a dedicated team of trained nurses, ensuring consistency across participants.

To minimize measurement bias, all outcome assessments were carried out by research nurses who were blinded to group allocation. The nurses recorded pain intensity using the Numerical Rating Scale (NRS) without any indication of the treatment arm. Consequently, the trial employed single blinding (outcome assessors blinded) while participants remained unblinded, and we acknowledge this as a limitation in the discussion.

### Intervention measures

Standardized Pharmacologic Management: Strictly adhere to a standardized Pregabalin regimen, initiating treatment at 75 mg twice daily for all patients. If the Numeric Rating Scale (NRS) pain score remains ≥5 for three consecutive days, the dose will be increased by 75 mg within two days, with a maximum dose not exceeding 300 mg per day. Conversely, if the NRS score is ≤3 for three consecutive days, the dose will be maintained or reduced by 75 mg. Efficacy and adverse reactions (e.g., somnolence, dizziness, weight gain) will be closely monitored, and therapy will be discontinued and switched to standard treatment if severe adverse reactions occur. Both groups received the same standardized drug treatment baseline.

Conventional nursing group: The control group will receive standard clinical care, which includes: (1) Condition Monitoring: closely observe changes in the patient’s condition, document the frequency, intensity, and nature of pain, and regularly assess the healing status of skin lesions; (2) Skin Care: maintain skin cleanliness to prevent secondary infection; (3) Health Education: provide information on postherpetic neuralgia and self-management education to enhance patients’ self-management abilities.

5A nursing group: the observation group implements the 5A nursing mode, which includes five key steps: (1) Assess: conduct in-depth interviews with patients, gather demographic and clinical information, as well as lifestyle data, to assess pain experience, lifestyle patterns, and quality of life; (2) Advise: provide disease manuals, educational videos, and regular lectures covering pain management, skin care, and chronic disease management, and offer personalized advice on diet, sleep, and exercise to address unhealthy habits; (3) Agree: assess pain and explain the treatment plan, with a focus on sleep quality, guiding patients to establish a calm and comfortable sleep environment; (4) Assist: assist patients in adhering to medication regimens, monitor efficacy and adverse reactions, and facilitate the receipt of physical therapies such as infrared therapy, acupuncture, moxibustion, and massage; (5) Arrange: develop a follow-up plan, conducting regular follow-ups via telephone, outpatient visits, or telemedicine to assess changes in pain and quality of life, and adjust the care plan promptly based on follow-up results. Supplementary Table 1 provides a detailed overview of the five steps of the 5A model, along with their respective implementation methods.

The Numeric Rating Scale (NRS) will be used to quantitatively assess pain levels in both groups at baseline, day 1, day 14, and one month post-intervention, facilitating a comparative analysis of the additional effect of the 5A nursing model on pain relief. After discharge, patients will be followed up by phone or outpatient visits.

### Data collection

To gather comprehensive information, the following categories of data were collected from patients suffering from postherpetic neuralgia: (1) Demographic Information: This included age, gender, height, weight, smoking habits, and alcohol consumption patterns. (2) Medical History: Information on the presence or absence of hypertension, diabetes, and coronary heart disease was documented. (3) Biochemical Parameters: The collection encompassed white blood cell count (WBC), neutrophil count (NEUT), and platelet count (PL). (4) Disease-Specific Information: Details such as the location of pain, duration of illness (specified in days and months), medication usage (including specific names, timing, and method of administration), occurrence of adverse drug reactions, and length of hospital stay were recorded. (5) Pain Assessment: The results of pain assessment were the primary outcome of the study. Pre- and post-intervention pain assessments using the Numeric Rating Scale (NRS) score (ranging from 0 to 10) were conducted (15). Specifically, NRS scores were collected at various time points, including before the intervention, 1 day, 14 days, and 1 month post-intervention. A cross-check of all “Adverse Event Record Forms” was conducted by two independent researchers. In the event of any discrepancy, the subjects will be re-examined to ascertain the veracity of the information provided.

### Sample size

Because the main analysis will use a mixed effects model (or ANCOVA) with baseline values as covariates, an approximate formula for two sample t-test considering repeated measures correlation can be used: $n=\frac{2(Z_{1\text{-}/2}+Z_{1\text{-}}{)}^{2}\;\times {\;}^{2}\;\times (1\text{-}r)}{2}$. According to IMMPACT recommendations (16) and Farrar et al.’s (17) systematic evaluation of changes in chronic pain NRS, a decrease of NRS≥1.5 points is considered the least clinically significant difference (MCID). Therefore, using *Δ* = 1.5, *α* = 0.05, efficacy = 0.80, two groups of SD ≈ 2.5, and baseline follow-up correlation coefficient r ≈ 0.5 for calculation, the required sample size for each group is about 42 (a total of 84), and 120 cases were actually enrolled (60 cases per group).

### Statistical analysis

SPSS 25.0 statistical software was used as the data analysis tool. The measurement data is expressed as mean ± standard deviation (SD). At baseline, independent sample t-test (for normally distributed data) or nonparametric rank sum test (for skewed distribution) is used to compare groups based on the distribution of the data; Count data characterized by their frequency (or ratio) and perform chi square tests to compare the categorical variables of each group. Further analysis was conducted on the patient's pain score (NRS) using a mixed design analysis of variance. Among them, group refers to inter group factors, and time refers to intra group factors. The Mauchly sphericity test was conducted on the repeated measurement factor ‘time’, and the results showed that the sphericity hypothesis did not hold (*P* < 0.05). Therefore, the subsequent analysis adopted the Greenhouse Geisser correction method. Perform simple effect analysis on significant interaction effects and use Bonferroni method for *post hoc* comparison. The statistical significance level is set to *α*=0.05.

## Results

A total of 120 patients with postherpetic neuralgia (PHN) who fulfilled the inclusion and exclusion criteria were invited to participate in this study. They were evenly divided into two groups: the conventional nursing group (60 patients) and the 5A nursing group (60 patients). Among the 120 participants, no mild, moderate, or severe adverse events occurred. The age variable is presented in terms of mean and standard deviation. Specifically, the routine nursing group exhibits an average age of 67.02 ± 10.98 years, whereas the intervention group demonstrates an average age of 64.88 ± 11.92 years. Notably, the statistical analysis reveals no significant difference in age between the two groups (*P* < 0.310). A comparison of the baseline general characteristics between the two groups, including gender, age, lifestyle, disease history, pain location, and blood parameters such as white blood cell (WBC) count and platelet (PL) count, revealed no statistically significant differences (*P* > 0.05) (Table 1). Descriptive statistical results showed that the pain scores of the intervention group showed a significant downward trend over time (pre intervention: 6.82 ± 1.50, intervention 30 days: 1.93 ± 0.94), while the control group had a smaller decrease (pre intervention: 6.77 ± 0.85, intervention 30 days: 3.03 ± 0.61) (Table 2).

**Table 1:** Comparison of baseline general information between two groups of patients.

Variable	Routine nursing group (*n* = 60)	5A Nursing Group (*n* = 60)	t/x2	*P*
General information data				
Age(year)	67.02 ± 10.98	64.88 ± 11.92	−1.019	0.310
Gender			0.845	0.462
Male	24 (40.0%)	31 (51.7%)		
Female	36 (60.0%)	29 (48.3%)		
Smoking			1.477	0.311
No	46 (76.7%)	40 (66.7%)		
Yes	14 (23.3%)	20 (33.3%)		
Drinking			1.087	0.404
No	42 (78.3%)	42 (70.0%)		
Yes	13 (21.7%)	18 (30.0%)		
BMI(kg/m2)	18.85 ± 1.72	18.93 ± 3.15	0.152	0.879
Past medical history				
Diabetes			2.342	0.189
No	43 (71.7%)	50 (83.3%)		
Yes	17 (28.3%)	10 (16.7%)		
Hypertension			0.586	0.566
No	37 (61.7%)	41 (68.3%)		
Yes	23 (38.3%)	19 (31.7%)		
Coronary heart disease			0.209	1.000
No	57 (95.0%)	58 (96.7%)		
Yes	3 (5.0%)	2 (3.3%)		
Biochemical indicators				
WBC(109/L)	5.92 ± 1.22	5.90 ± 1.14	−0.188	0.907
Neutrophils(109/L)	1.06 ± 1.32	0.71 ± 0.61	−1.826	0.071
PL(109/L)	209.78 ± 35.60	234.60 ± 37.82	0.944	0.349
Disease information				
Pain area			5.200	0.146
Head	3 (5.0%)	2 (3.3%)		
Back	44 (73.3%)	52 (86.7%)		
Abdomen	1 (1.7%)	2 (3.3%)		
Limbs	12 (20.0%)	4 (6.7%)		
NRS scores	6.77 ± 0.85	6.82 ± 1.50	0.224	0.823

BMI, body mass index; WBC, white blood cell count; PL, platelet count; NRS score, numeric rating scale score.

**Table 2:** Pain scores of intervention group and control group before and after intervention.

Variable	Routine nursing group (*n* = 60)	5A Nursing Group (*n* = 60)
NRS score		
Before intervention	6.77 ± 0.85	6.82 ± 1.50
First day after intervention	6.00 ± 0.84	6.05 ± 1.31
14 days after intervention	4.02 ± 0.95	2.68 ± 0.62
1 month after intervention or at discharge	3.03 ± 0.61	1.93 ± 0.94

NRS score, numeric rating scale score; Control group, routine nursing group; Intervention group, 5A nursing group.

The results of the mixed design analysis of variance show that the time main effect is significant, F = 978.014, *P* < 0.001, Partial *η*2 = 0.892. The main effect of the group is significant, F = 17.087, *P* < 0.001, Partial *η*2 = 0.126. The interaction between time and group is significant, F = 31.020, *P* < 0.001, Partial *η*2 = 0.208 (Table 3). Due to the significant interaction, we conducted a simple effects analysis. Inter group comparison: There was no significant difference in pain scores between the two groups of patients before intervention and on the first day after intervention (*P* > 0.05); On the 14th and 30th day after intervention, the pain scores of the intervention group were significantly lower than those of the control group (all *P* < 0.05) (Table 4); Intra group comparison: For the intervention group, there were significant differences (all *P* < 0.05) in pain scores between the four time points, indicating a significant decrease in pain scores as the intervention continued. The results showed that compared with the control group, the intervention measures could significantly and continuously reduce patients’ pain scores, and the intervention effect increased over time.

**Table 3:** Testing of inter subject and inter subject effects.

Variable	Repeated measurement *F*-test		
	F	*P*	Partial η²
Time	978.014	<0.001	0.892
Group	17.087	<0.001	0.126
Time × Group	31.020	<0.001	0.208

**Table 4:** Comparison of NRS scores between intervention groups and control group at four time points.

Time	Mean value difference	Standard error	*P*	95%CI
Before intervention	0.05	0.223	0.823	(−0.391, 0.491)
First day after intervention	0.05	0.201	0.804	(−0.348, 0.448)
14 days after intervention	−1.333	0.146	<0.001	(−1.623, −1.043)
1 month after intervention or at discharge	−1.100	0.144	<0.001	(−1.386, −0.814)

## Discussion

For individuals suffering from PHN, they frequently confront persistent or intermittent severe pain, exerting profound effects on their physical well-being (18, 19). As societal quality of life advances, the significance of pain management has garnered heightened attention from both patients and healthcare professionals (20, 21). The persistence of diverse pain types among patients can induce a multitude of detrimental effects on their physiological, psychological, and behavioral facets, potentially leading to heightened readmission rates, extended hospital stays, and diminished patient satisfaction (22). This randomized controlled trial demonstrated that nursing interventions based on the 5A model led to statistically significant and clinically meaningful reductions in pain intensity among patients with PHN, outperforming conventional care. At 14 days and 1 month post-intervention, NRS scores in the 5A group were significantly lower than those in the control group (*P* < 0.05), with mean differences exceeding the 1.5-point minimum clinically important difference (MCID) recommended. These findings indicate that the 5A model is not only statistically superior but also clinically relevant for the management of PHN-related pain.

The primary contribution of this study lies in elucidating the potential mechanisms through which the 5A model alleviates pain in patients with postherpetic neuralgia (PHN). PHN is characterized by peripheral and central sensitization, and is frequently accompanied by anxiety, sleep disturbances, and poor medication adherence (3, 4). The 5A model addresses these multidimensional issues synergistically through its structured five-step process (Assess, Advise, Agree, Assist, Arrange).

The step of “Agree” plays a crucial role in enhancing self-efficacy and treatment compliance. PHN patients often feel frustrated due to persistent pain and complex medication regimens. By involving patients in goal setting and nursing plan development, ‘reaching consensus’ cultivates patients’ sense of ownership and commitment. This psychological empowerment has been shown to activate the descending inhibitory pain pathway and reduce pain catastrophizing (23). ElSobky et al. (24). found in their research on children with diabetes that the collaborative goal setting of the 5A model significantly improved self-management behavior, which is consistent with our observation that patients’ participation in pain management increased.

The “Assist” step directly targets barriers to medication adherence, a critical component given that first-line pharmacotherapies for PHN—such as pregabalin—are frequently associated with dose-dependent adverse reactions, including dizziness and somnolence (6, 7). In the intervention protocol, nurses in the 5A group proactively monitored for side effects, offered coping strategies, and communicated with physicians to facilitate timely dose adjustments. This proactive support likely contributed to reduced treatment interruptions and optimized analgesic outcomes. Heidari et al. (10) provide further support for this mechanism, reporting that the 5A model improved clinical outcomes in patients with chronic obstructive pulmonary disease by enhancing adherence to complex treatment regimens.

The “Assess” and “Arrange” steps ensure dynamic and personalized pain management. As PHN pain is known to fluctuate over time, a static, one-size-fits-all intervention is often insufficient (20). The initial comprehensive assessment enabled the identification of individual pain triggers, lifestyle factors, and psychological comorbidities, thereby enabling the development of personalized recommendations regarding sleep hygiene, stress reduction, and physical therapy. Furthermore, structured follow-up (Arrange) facilitated the timely identification of pain exacerbations or adverse reactions, allowing for prompt adjustment of the nursing plan. This dynamic feedback loop aligns with the chronic disease care model, which emphasizes proactive and planned interactions to improve long-term outcomes (25).

Our findings align with those of studies examining the 5A model in other chronic patient populations. Keivan et al. (23) demonstrated that a 5A-based self-management plan improved cognitive function and sleep quality in hemodialysis patients. Similarly, Zhang et al. (26) reported that the 5A model enhanced self-efficacy and reduced cancer-related fatigue in patients undergoing postoperative chemotherapy for liver cancer. However, the present study extends this body of evidence by linking specific components of the 5A model to mechanisms relevant to neuropathic pain management, including improved medication adherence and enhanced psychological coping.

It is worth noting, however, that not all studies have reported positive outcomes with the 5A model. Moradi et al. (27) found that although a 5A-based intervention improved quality-of-life scores in elderly patients with hypertension, the improvements did not reach statistical significance. This discrepancy may be attributed to differences in study populations, intervention duration, or outcome measures. In the current study, the relatively short follow-up period (one month) may have captured initial benefits associated with enhanced adherence and support; nonetheless, the long-term effects on pain recurrence or quality of life remain to be established.

## Limitations and future directions

This study has several limitations. First, the follow-up period was limited to one month, precluding assessment of the long-term sustainability of pain relief or the likelihood of pain recurrence. Future research should extend the follow-up duration to 6 or 12 months to evaluate the persistence of treatment effects. Second, this was a single-center study with a relatively modest sample size (*N* = 120). Although randomization ensured baseline comparability, the generalizability of our findings to other populations or clinical settings remains limited. Larger, multi-center trials with more diverse samples are warranted. Third, despite efforts to blind outcome assessors, the nature of the intervention precluded blinding of participants to their group allocation, which may have introduced performance bias. Future studies may consider incorporating an active control group that receives equivalent attention to control for potential Hawthorne effects.

Despite these limitations, this study provides the first evidence that nursing interventions based on the 5A model can significantly alleviate pain in patients with PHN. By elucidating the mechanistic links between specific 5A steps and PHN pathophysiology, our findings offer a theoretical foundation for integrating this model into routine clinical practice.

## Conclusion

The results of this study indicate that implementing the 5A nursing model has the potential to improve pain management in patients with postherpetic neuralgia (PHN). Therefore, it is recommended that clinical nursing staff use this model as a strategy for managing patient pain and strengthening intervention measures to promote patient health and recovery.

## Data Availability

The original contributions presented in the study are included in the article/Supplementary Material, further inquiries can be directed to the corresponding author.

## References

[1] Nair PA, Patel BC. Herpes zoster. In: *StatPearls [Internet]*. Treasure Island (FL): StatPearls Publishing (2026). Available online at: https://www.ncbi.nlm.nih.gov/books/NBK441824/

[2] Van Oorschot D Vroling H Bunge E Diaz-Decaro J Curran D Yawn B. A systematic literature review of herpes zoster incidence worldwide. Hum Vaccin Immunother. (2021) 17(6):1714–32. 10.1080/21645515.2020.1847582 33651654 PMC8115759

[3] Mizukami A Sato K Adachi K Matthews S Holl K Matsuki T. Impact of herpes zoster and post-herpetic neuralgia on health-related quality of life in Japanese adults aged 60 years or older: results from a prospective, observational cohort study. Clin Drug Investig. (2018) 38(1):29–37. 10.1007/s40261-017-0581-5 29086340 PMC5762777

[4] Li X Chen P He J Huang X Tang D Chen L. Comparison of the efficacy and safety of temporary spinal cord stimulation versus pulsed radiofrequency for postherpetic neuralgia: a prospective randomized controlled trial. Pain Res Manag. (2022) 2022:3880424. 10.1155/2022/3880424 36267666 PMC9578922

[5] Yang F Yu S Fan B Liu Y Chen YX Kudel I. The epidemiology of herpes zoster and postherpetic neuralgia in China: results from a cross-sectional study. Pain Ther. (2019) 8(2):249–59. 10.1007/s40122-019-0127-z 31218562 PMC6857181

[6] Lin CS Lin YC Lao HC Chen CC. Interventional treatments for postherpetic neuralgia: a systematic review. Pain Physician. (2019) 22(3):209–28. 10.36076/ppj/2019.22.209 31151330

[7] Lee SH Lee JY Yeon H Rho MC Bae J Park HJ. Pain changes and new neurologic sign in post-herpetic neuralgia: a clue in the diagnosis of malignancy-a case report. Ann Palliat Med. (2022) 11(8):2773–7. 10.21037/apm-21-2567 35073719

[8] Lee S Hwang Y Lim H. 5A’s Behavior change model improves nutrition knowledge and intake among adolescent athletes. Clin Nutr Res. (2024) 13(4):244–56. 10.7762/cnr.2024.13.4.244 39526209 PMC11543449

[9] Rokni S, Rezaei Z, Noghabi AD, Sajjadi M, Mohammadpour A. Evaluation of the effects of diabetes self-management education based on 5A model on the quality of life and blood glucose of women with gestational diabetes mellitus: an experimental study in eastern Iran. J Prev Med Hyg. (2022) 63(3):E442–7. 10.15167/2421-4248/jpmh2022.63.3.2611 36415299 PMC9648546

[10] Heidari M Fayazi S Borsi SH Latifi M Moradbeigi K Torghi MT. Effect of the 5A model on clinical Status indexes of COPD patients. Rehabil Nurs. (2018) 43(3):158–66. 10.1097/rnj.0000000000000012 29710060

[11] Xu P Zheng W Zhu Y. Effect analysis of lung rehabilitation training in 5A nursing mode for elderly patients with COPD based on x-ray. Comput Math Methods Med. (2022) 2022:1963426. 10.1155/2022/1963426 35734776 PMC9208961

[12] Zaremobini F Farajzadegan Z Kazemi A Salehi M. Effect of using 5A’s model for lifestyle counseling on psychological symptoms in women with polycystic ovary syndrome: a randomized field trial. Sci Rep. (2022) 12(1):21847. 10.1038/s41598-022-26274-z 36528714 PMC9759549

[13] Guerra PH, Sposito LAC, da Costa FF, Fermino RC, Papini CB, Rech CR. Effectiveness of the 5A counseling model-based interventions on physical activity indicators in adults: a systematic review. Behav Sci (Basel). (2023) 13(6):476. 10.3390/bs13060476 PMC10295221 37366729

[14] Postherpetic neuralgia. In *UpToDate*. Waltham, MA: Wolters Kluwer (2026). Available online at: https://www. uptodate.com/contents/postherpetic-neuralgia (Accessed May 17, 2026)

[15] Wadhwa A Akash VS Bharadwaj S Kadarapura NG Konar SK Naik S. Association between patient characteristics and dissatisfaction after cranial neurosurgery: a prospective observational study. J Neurosci Rural Pract. (2023) 14(2):280–5. 10.25259/JNRP_31_2023 37181196 PMC10174114

[16] Dworkin RH Turk DC Wyrwich KW Beaton D Cleeland CS Farrar JT. Interpreting the clinical importance of treatment outcomes in chronic pain clinical trials: iMMPACT recommendations. J Pain. (2008) 9(2):105–21. 10.1016/j.jpain.2007.09.005 18055266

[17] Farrar JT, Young JP Jr, LaMoreaux L, Werth JL, Poole MR. Clinical importance of changes in chronic pain intensity measured on an 11-point numerical pain rating scale. Pain. (2001) 94(2):149–58. 10.1016/S0304-3959(01)00349-9 11690728

[18] Jiang X Kuang H Lv H Xiong J Li J Hong S. Aberrant functional and causal connectivity of the amygdala in herpes zoster and post-herpetic neuralgia patients. Br J Radiol. (2023) 96(1152):20230338. 10.1259/bjr.20230338 37750852 PMC10646639

[19] Koshy E Mengting L Kumar H Jianbo W. Epidemiology, treatment and prevention of herpes zoster: a comprehensive review. Indian J Dermatol Venereol Leprol. (2018) 84(3):251–62. 10.4103/ijdvl.IJDVL_1021_16 29516900

[20] Hadley GR Gayle JA Ripoll J Jones MR Argoff CE Kaye RJ. Post-herpetic neuralgia: a review. Curr Pain Headache Rep. (2016) 20(3):17. 10.1007/s11916-016-0548-x 26879875

[21] Schutzer-Weissmann J Farquhar-Smith P. Post-herpetic neuralgia - a review of current management and future directions. Expert Opin Pharmacother. (2017) 18(16):1739–50. 10.1080/14656566.2017.1392508 29025327

[22] Werner RN Ghoreschi K. Herpes zoster-prevention, diagnosis, and treatment. Hautarzt. (2022) 73(6):442–51. 10.1007/s00105-022-04992-9 35477786

[23] Keivan S Shariati A Miladinia M Haghighizadeh MH. Role of self-management program based on 5A nursing model in quality of life among patients undergoing hemodialysis: a randomized clinical trial. BMC Nephrol. (2023) 24(1):58. 10.1186/s12882-023-03108-2 36922765 PMC10017059

[24] Elsobky FA Darweesh HAM Alzahrani SHA Bassam SEA. The impact of a self-management program based on the 5 a’s model on type 1 diabetes in school-aged children. Ann Nutr Metab. (2022) 78(4):197–206. 10.1159/000524590 35671742

[25] Goodarzi F Mojahed S Zaremobini F Sarebanhassanabadi M. Effect of using 5A’s model for self-management counseling on quality of life and self-efficacy in women with polycystic ovary syndrome: a randomized clinical trial. Iran J Nurs Midwifery Res. (2025) 30(6):832–8. 10.4103/ijnmr.ijnmr_12_24 41311600 PMC12655837

[26] Zhang X, Lai M, Wu D, Luo P, Fu S. The effect of 5A nursing intervention on living quality and self-care efficacy of patients undergoing chemotherapy after hepatocellular carcinoma surgery. Am J Transl Res. (2021) 13(6):6638–45. 34306407 PMC8290751

[27] Moradi M Nasiri M Jahanshahi M Hajiahmadi M. The effects of a self-management program based on the 5 a’s model on self-efficacy among older men with hypertension. Nursing Midwifery Studies. (2019) 8(1):21–7. 10.4103/nms.nms_97_17

