# Supplementary material for: The impact of the 5A nursing model on pain in patients with postherpetic neuralgia: a randomized controlled trial
Source: Front Pain Res (Lausanne). 2026 Jun 17;7:1769480. doi: 10.3389/fpain.2026.1769480 (PMC13319014; doi:10.3389/fpain.2026.1769480)
Supplement: Supplementary file 1 [file Table1.docx]

Table S1. 5A nursing model steps for patients with postherpetic neuralgia

| Item | Content |
| --- | --- |
| Assess | Physicians and nurses specializing in the pain management department conducted in-depth, face-to-face interviews with patients, meticulously gathering demographic details, comprehensive clinical information (encompassing disease manifestations, surgical interventions, dietary practices, and exercise routines), and subsequently compiling detailed case files. These efforts aimed at fostering a holistic understanding of each patient's pain experience, lifestyle patterns, quality of life, and their capacity for self-management and self-efficacy. |
| Advise | **Knowledge Support:** We offer extensive resources to patients, including disease manuals, educational videos, and regular lectures. Topics cover pain management, skin care, and chronic disease management. We offer individual and group sessions to suit diverse learning needs. **Lifestyle Changes:** We identify unhealthy habits through assessments, educate patients on risks, and provide personalized advice on diet, sleep, and exercise to reduce pain and speed recovery. **Self-Management Training:** Patients learn skills like pain monitoring, medication adherence, and relaxation. We tailor sessions to individual needs and send educational content via SMS and WeChat. **Psychological Support:** We prioritize patients' emotional well-being, offering counseling, relaxation training, and cognitive-behavioral therapy to build resilience and improve quality of life during treatment. |
| Agree | **Pain Management:** We evaluate pain, explain causes, treatments, and consequences. We promote physical activity and mental relaxation, address psychological factors, and engage patients in activities to distract from pain. Analgesics are administered as needed, with education on pain management tools like pumps and patches. **Skin Care:** we educate patients and their families on pain management, including the use of pain pumps and patches, elucidating drug mechanisms, adverse reaction monitoring, and enhancing treatment adherence. **Sleep Care:** For postherpetic neuralgia patients, we assess sleep quality, create a calm environment, and establish pre-sleep routines to improve sleep. **Medication Administration:** We guide patients on analgesic use, monitor for adverse reactions, and observe for sensory disturbances. **Health Education:** We inform patients about the nursing plan, create a comfortable medical environment, explain the impact of chronic diseases, provide disease knowledge and prevention tips, and assist in establishing healthy lifestyles. |
| Assist | **Implementation Content:** Medication Therapy Assistance: We ensure that patients adhere to their medication schedules, consuming the prescribed dosages on time. Our vigilant monitoring of drug efficacy and adverse reactions allows for prompt adjustments to the nursing plans as necessary.  Physical Therapy Assistance: We actively facilitate patients in receiving physical therapy treatments, encompassing infrared therapy, acupuncture and moxibustion, massage, and other relevant modalities. **Psychological Intervention Assistance:** We provide patients with comprehensive psychological counseling and intervention services, aiming to address their emotional needs and foster mental well-being. **Self-Management Support:** Through regular assessments of patients' self-management abilities, we offer tailored guidance and support, empowering them to adopt healthy lifestyles and behavioral patterns. |
| Arrange | **Regular Follow up:** Develop a follow-up plan and regularly follow up with patients through telephone, outpatient, or remote medical methods to understand their pain changes, treatment effectiveness, and quality of life. **Effect evaluation:** Evaluate the nursing effect based on the follow-up results, and adjust the treatment plan and nursing plan in a timely manner. **Continuous support:** Provide patients with continuous support and assistance, including pain management, psychological support, lifestyle guidance, etc., to ensure that patients receive comprehensive care and attention throughout the entire treatment process. |
